# Evaluation of body shape as a human body composition assessment in isolated conditions and remote environments

**DOI:** 10.1038/s41526-024-00412-5

**Published:** 2024-06-24

**Authors:** Michael C. Wong, Jonathan P. Bennett, Lambert T. Leong, Yong E. Liu, Nisa N. Kelly, John Cherry, Kate Kloza, Bosco Li, Sandra Iuliano, Jean Sibonga, Aenor Sawyer, Jeff Ayton, John A. Shepherd

**Affiliations:** 1 Department of Epidemiology, University of Hawaii Cancer Center, Honolulu, HI USA; 2 Polar Medicine Unit, Australian Antarctic Division, Kingston, Australia; 3 Departments of Medicine and Endocrinology, Austin Health, University of Melbourne, Melbourne, Australia; 4 National Aeronautics and Space Administration Johnson Space Center, Houston, TX USA; 5 UC Space Health, Dept of Orthopedic Surgery, University of California, San Francisco, CA USA

**Keywords:** Three-dimensional imaging, Nutrition

## Abstract

Individuals in isolated and extreme environments can experience debilitating side-effects including significant decreases in fat-free mass (FFM) from disuse and inadequate nutrition. The objective of this study was to determine the strengths and weaknesses of three-dimensional optical (3DO) imaging for monitoring body composition in either simulated or actual remote environments. Thirty healthy adults (ASTRO, male = 15) and twenty-two Antarctic Expeditioners (ABCS, male = 18) were assessed for body composition. ASTRO participants completed duplicate 3DO scans while standing and inverted by gravity boots plus a single dual-energy X-ray absorptiometry (DXA) scan. The inverted scans were an analog for fluid redistribution from gravity changes. An existing body composition model was used to estimate fat mass (FM) and FFM from 3DO meshes. 3DO body composition estimates were compared to DXA with linear regression and reported with the coefficient of determination (*R*^*2*^) and root mean square error (RMSE). ABCS participants received only duplicate 3DO scans on a monthly basis. Standing ASTRO meshes achieved an *R*^*2*^ of 0.76 and 0.97 with an RMSE of 2.62 and 2.04 kg for FM and FFM, while inverted meshes achieved an *R*^*2*^ of 0.52 and 0.93 with an RMSE of 2.84 and 3.23 kg for FM and FFM, respectively, compared to DXA. For the ABCS arm, mean weight, FM, and FFM changes were −0.47, 0.06, and −0.54 kg, respectively. Simulated fluid redistribution decreased the accuracy of estimated body composition values from 3DO scans. However, FFM stayed robust. 3DO imaging showed good absolute accuracy for body composition assessment in isolated and remote environments.

## Introduction

Astronauts and Antarctic expeditioners, who live in isolated conditions and remote environments for long durations, experience physiological adaptations that can negatively impact their long-term health. While in microgravity, astronauts can lose over 30% of skeletal muscle mass in under 6 months^1^ as well as areal bone mineral density loss of 0.9%/ month at the spine and 1.5%/ month at the hip^2^. These drastic physiological changes share similar frailty and shape characteristics of sarcopenia^3^ and may lead to premature osteoporosis when returning to Earth^4^. Antarctic expeditioners may have limited capacities for physical activity and adequate nutrition as a result of prolonged isolation, confinement, and extreme Antarctic weather that mimics conditions of long duration isolation similar to that of spaceflight^5–7^. Given these specific challenges, it is important to monitor changes in fat mass (FM) and fat-free mass (FFM), to enable the implementation of appropriate countermeasures.

Body composition surrogates such as body mass index (BMI), weight, and anthropometry (e.g. waist and hip circumference) have been used in these environments^6,8^. However, it is important to acknowledge their limitations as they fail to offer comprehensive data on total or regional FM and FFM. Without a mode to assess body composition in these remote areas, the opportunities to make modifications are limited and a potential barrier for longer missions (e.g., Lunar and Mars missions and other remote and austere terrestrial healthcare environments).

Body composition modalities such as: dual-energy X-ray absorptiometry (DXA)^9^, bioelectrical impedance analysis (BIA)^10^, and air displacement plethysmography (ADP)^11^ are commonly used to quantify body composition in clinical and laboratory settings. However, these methods have limitations including the size and weight of equipment, need for qualified technicians (DXA and ADP), or modeling assumptions that do not hold true due to fluid redistribution (BIA)^10,12^, that make them less than desirable for remote area application. The ideal technique needs to be light, compact, account for fluid redistribution, and provide regional body composition estimates. As changes in FM and FFM lead to body shape changes, shape may be the intuitive predictor for body composition.

Recent advances in three-dimensional optical (3DO) imaging and body composition modeling offer the possibility of using body shape to predict body composition in remote and isolated environments. 3DO can identify signals associated with muscle, bone, and frailty^13^. 3DO scanners use depth cameras to scan the surface of the body to provide accurate and precise automated anthropometry and a high-resolution 3D mesh^14,15^, which is used to create statistical shape models to predict body composition^13,16–19^. A recent paper showed that reposing these meshes to a standardized pose before analysis improved the accuracy and precision regardless of initial pose^20^. In terms of applicability in remote environments, this is important as the user may not be able to get into manufacturer-recommended positioning as they are free-floating or in confined spaces. However, it is still unknown if current 3DO models can account for fluid redistribution in simulated microgravity or if remote users can acquire valid scans with limited training^21^. This study aimed to evaluate the use of 3DO imaging to assess body composition in simulated or remote and isolated environments. We hypothesize that 3DO can provide accurate and precise body composition estimates for these populations and remote users can successfully acquire valid data.

## Results

### Participant statistics

There was a total of 52 participants in this study (Table 1). ASTRO consisted of 30 participants (50% male) with a mean age and BMI of 31 years and 25.6 kg m^-2, respectively. ABCS consisted was 22 participants (4 females); the average age and BMI were 44 years and 27.8 kg m^-2, respectively. Although 3DO body composition estimates were derived from sex-specific models^20^, results from this study were presented with males and females combined due to the small sample sizes.

**Table 1:** Sample Characteristics

	ASTRO Females (*N* = 15)	ASTRO Males (*N* = 15)	ABCS (*N* = 22, 4 Female)
**Age (years)**			
Mean (SD)	31.1 (9.58)	31.4 (8.35)	43.9 (10.7)
Min, Max	23.1, 59.8	20.3, 51.5	29.0, 62.0
**Height (cm)**			
Mean (SD)	162 (6.85)	176 (7.50)	176.9 (5.8)
Min, Max	149, 178	161, 187	167.4, 203.0
**Weight (kg)**			
Mean (SD)	64.4 (9.33)	83.0 (13.5)	87.2 (14.9)
Min, Max	46.9, 79.7	56.2, 106	62.3, 119.6
**BMI** **(kg m^-2)**			
Mean (SD)	24.4 (2.81)	26.8 (3.15)	27.8 (3.9)
Min, Max	20.2, 30.1	21.7, 31.2	17.8, 32.2
**Ethnicity**			
Asian	9 (60.0%)	9 (60.0%)	
Hispanic	1 (6.7%)	0 (0%)	
NHOPI	0 (0%)	1 (6.7%)	
White	5 (33.3%)	5 (33.3%)	
**DXA Total FM (kg)**			
Mean (SD)	17.0 (5.29)	13.4 (5.31)	
Min, Max	10.5, 26.0	7.25, 25.7	
**DXA Total FFM (kg)**			
Mean (SD)	47.6 (6.05)	70.0 (10.7)	
Min, Max	36.7, 57.3	48.9, 85.3	
**DXA Percent Fat** **(%)**			
Mean (SD)	28.4 (5.42)	18.0 (5.16)	
Min, Max	20.5, 37.6	11.4, 27.1	
**DXA VAT (kg)**			
Mean (SD)	0.18 (0.12)	0.29 (0.12)	
Min, Max	0.08, 0.52	0.15, 0.60	

*BMI* body mass index, *DXA* dual-energy X-ray absorptiometry, *FM* fat mass, *FFM* fat-free mass, *VAT* visceral adipose tissue.

### 3D optical accuracy

The SRL-3 scanner had a strong agreement with criterion optical scanner, Fit3D ProScanner, for all body composition variables (*R*^*2*^*s*: 0.77–0.99; RMSEs: 0.04–1.9 kg) (Table 2). Although the agreement was slightly lower when compared to DXA (*R*^*2*^*s*: 0.54–0.97; RMSEs: 0.06–3.42 kg), the SRL-3 achieved similar results to the Fit3D vs. DXA comparison (*R*^*2*^*s*: 0.51–0.96; RMSEs: 0.07–4.07) and had better agreement to DXA than Fit3D in some cases. The inverted scans had a strong agreement for total FFM to the SRL-3, Fit3D, and DXA (*R*^*2*^*s*; 0.95, 0.95, 0.93; RMSEs; 2.71 kg, 2.80 kg, and 3.23 kg, respectively) as well as other regional FFM estimates. The total FM estimate on the inverted scans had a lower agreement to the SRL-3, Fit3D, and DXA (*R*^*2*^*s*; 0.77, 0.70, 0.52; RMSEs; 1.96 kg, 2.24 kg, and 2.84 kg, respectively). However, the RMSEs remained comparable to other comparisons (i.e., Fit3D vs DXA and 3-camera scanner vs DXA). The other regional fat estimates of the inverted scans demonstrated a similar trend. The SRL-1 scanner performed similarly as the 3-camera scanner when compared to DXA (*R*^2^s: 0.49–0.95; RMSEs: 0.07–4.03) and the Fit3D (*R*^2^s: 0.49–0.95; RMSEs: 0.07–4.03) (Table 3).

**Table 2:** Comparison between SRL-3 and inverted scans to criterion measures (*n* = 30)

Outcome	SRL-3 vs DXA *R*^2^	SRL-3 vs DXA RMSE	Fit3D vs DXA *R*^2^	Fit3D vs DXA RMSE	SRL-3 vs Fit3D *R*^2^	SRL-3 vs Fit3D RMSE	SRL-3, standing vs Inverted *R*^2^	SRL-3, standing vs Inverted RMSE	Fit3D standing vs SRL-3 Inverted *R*^2^	Fit3D standing vs SRL-3 Inverted RMSE	DXA vs SRL-3 Inverted *R*^2^	DXA vs SRL-3 Inverted RMSE
Total FM	0.76	2.62	0.77	2.67	0.90	1.72	0.77	1.96	0.70	2.24	0.52	2.84
Total FFM	0.97	2.04	0.96	2.55	0.99	1.18	0.95	2.71	0.95	2.80	0.93	3.23
Percent Fat	0.70	3.42	0.71	4.07	0.91	1.90	0.64	2.87	0.73	2.47	0.50	3.35
VAT	0.78	0.06	0.68	0.07	0.89	0.04	0.43	0.10	0.30	0.11	0.39	0.11
Arm FM	0.54	0.26	0.51	0.28	0.86	0.14	0.64	0.20	0.60	0.21	0.21	0.29
Arm FFM	0.94	0.32	0.95	0.28	0.98	0.16	0.95	0.29	0.97	0.24	0.93	0.34
Leg FM	0.62	0.58	0.69	0.59	0.77	0.45	0.56	0.55	0.38	0.65	0.18	0.75
Leg FFM	0.92	0.62	0.93	0.58	0.98	0.35	0.93	0.51	0.95	0.41	0.86	0.69
Trunk FM	0.81	1.27	0.82	1.23	0.94	0.72	0.78	1.02	0.64	1.29	0.61	1.36
Trunk FFM	0.94	1.50	0.95	1.36	0.98	0.82	0.95	1.21	0.97	0.93	0.94	1.32

*FM* fat mass, *FFM* fat-free mass, *VAT* visceral adipose tissue.

All outcomes are presented in kg except percent fat.

**Table 3:** Comparison between SRL-1 and SRL-3 systems (standing) to criterion measures (*n* = 16)

Outcome	SRL-1 vs DXA *R*^2^	SRL-1 vs DXA RMSE	SRL-3 vs DXA *R*^2^	SRL-3 vs DXA RMSE	SRL-1 vs Fit3D *R*^2^	SRL-1 vs Fit3D RMSE	SRL-3 vs Fit3D *R*^2^	SRL-3 vs Fit3D RMSE
Total Fat	0.78	2.64	0.75	2.84	0.94	1.37	0.91	1.74
Total Lean	0.95	2.44	0.95	2.32	0.99	1.32	0.99	1.10
Percent Fat	0.60	4.03	0.57	3.93	0.92	1.77	0.92	1.73
VAT	0.60	0.07	0.72	0.07	0.92	0.03	0.88	0.05
Arm Fat	0.49	0.30	0.51	0.31	0.93	0.11	0.87	0.16
Arm Lean	0.94	0.28	0.90	0.37	0.98	0.16	0.98	0.15
Leg Fat	0.70	0.62	0.63	0.64	0.93	0.29	0.78	0.49
Leg Lean	0.91	0.67	0.90	0.64	0.99	0.25	0.95	0.43
Trunk Fat	0.85	1.10	0.84	1.19	0.95	0.61	0.94	0.75
Trunk Lean	0.92	1.62	0.92	1.57	0.99	0.43	0.97	0.98

*SRL* Shepherd Research Lab, *VAT* visceral adipose tissue.

All outcomes are presented in kg except percent fat.

### 3D optical test-retest precision

The SRL-3’s test-retest precision (%CVs: 0.97–6.62%; RMSEs: 0.02–0.75) performed similarly to the Fit3D (%CVs: 0.78–5.19%; RMSEs: 0.01–0.61) (Table 4), however, Fit3D’s precision performed slightly better overall. The precision of the inverted meshes were poor for all the FM metrics. However, all FFM metrics were comparable to the SRL-3 and Fit3D (%CVs; 2.3, 2.95, 1.34, 1.55; RMSEs; 1.32, 0.12, 0.14, and 0.47 kg). The SRL-1 had similar precision compared to the SRL-3 and Fit3D except for arm FM, which had a slightly higher %CV; 7.1%. After removal of failed scans (~ 20%), the SRL-1 precision from ABCS for total FM and FFM had nearly identical precision compared to the ASTRO SRL-1, which was in a more controlled laboratory setting.

**Table 4:** 3D optical test-retest precision (*n* = 30)

	SRL-3%CV	SRL-3 RMSE	Fit3D %CV	Fit3D RMSE	SRL-3 Inverted %CV	SRL-3 Inverted RMSE	SRL-1%CV	SRL-1 RMSE	ABCS %CV	ABCS RMSE
Total FM	2.83	0.54	2.39	0.43	7.35	1.32	3.82	0.76	0.71	0.73
Total FFM	0.97	0.54	0.78	0.43	2.30	1.32	1.44	0.76	1.50	0.78
Percent Fat	NA	0.75	NA	0.61	NA	1.99	NA	1.34	NA	0.89
VAT	6.62	0.02	5.19	0.01	14.9	0.04	6.43	0.02		
Arm FM	4.47	0.05	4.43	0.05	9.40	0.11	7.09	0.09		
Arm FFM	2.92	0.11	1.94	0.07	2.95	0.12	1.17	0.04		
Leg FM	4.42	0.16	3.20	0.11	9.00	0.32	4.33	0.17		
Leg FFM	2.07	0.20	1.12	0.10	1.34	0.14	1.69	0.17		
Trunk FM	2.24	0.19	2.16	0.17	6.86	0.56	2.40	0.21		
Trunk FFM	1.83	0.52	0.79	0.22	1.55	0.47	1.33	0.37		

*%CV* coefficient of variation, *FM* fat mass, *FFM* fat-free mass, *RMSE* root mean square error, *VAT* visceral adipose tissue.

All outcomes are presented in kg except percent fat.

SRL-1 (*n* = 15).

ABCS (*n* = 22).

### Antarctica

After confirming the successful capturing of reliable scan data, changes in body weight and composition from ABCS are presented in Table 5. Despite an overall weight stability by the group across the study period, ranging 2–5 months (−0.47 kg), changes ranged from a loss of 3.6 kg to a gain of 4.8 kg for total FM. Changes in total FFM ranged from a loss of 2.2 kg to a gain of 3.8 kg.

**Table 5:** Change in body weight and body composition parameters across testing periods in ABCS (*n* = 22, 4 female)

Variable	Average Δ	Min	Max	Percent Δ	Min	Max
Weight (kg)	−0.47	−0.10	−4.10	−0.62	−0.12	−4.56
FFM (kg)	0.06	0.04	3.80	0.01	0.07	−6.07
FM (kg)	−0.54	−0.02	4.82	−1.84	−0.11	16.9
Percent Fat	−0.41	0.52	3.62			

Δ (change), *FFM* (fat-free mass), *FM* (fat mass).

Weight was measured by scale. Body composition was estimated by 3DO.

### Supplementary Tables

The regional removal analysis showed the meshes with predicted regions performed similarly to the SRL-3 reference mesh (Supplementary Table 1). There were slight differences in anthropometry between the reference mesh and their regional removal counterparts (e.g. neck and arm circumference in the mesh with all parts removed) (Supplementary Table 2). However, most anthropometry from the regions removed were non-significant (*p* > 0.05). When comparing the inverted and standing meshes, the inverted meshes had significantly smaller waist, hip, and thigh circumferences, while having larger chest and arm circumferences (*p* < 0.034).

Additional scans were taken to evaluate the accuracy of 3DO with an 8 and 30 s rotation, respectively (Supplementary Table 3). These scans were compared to the standard SRL-3 mesh taken at 15 s. Overall, the agreement was strong for the 8 s (*R*^*2*^*s*: 0.88–0.99, RMSE: 0.05–1.52) and 30 s (*R*^*2*^*s*: 0.94–0.99, RMSE: 0.03–1.17) rotation speed scans.

## Discussion

3DO was evaluated as a potential body composition modality in-flight and for applicability in a remote setting with a confined testing space and limited resources. The SRL-3 and SRL-1 scanners were compared to criterion measures, Fit3D and DXA, and showed good accuracy and precision. The body composition estimates from scans with different rotation speeds agreed well with our reference (SRL-3, standing upright at 8 rpm). The inverted scans showed good overall agreement with the standing SRL-3 scans, the Fit3D, and the DXA’s FFM estimates. The SRL-1 system used for ABCS supported the use of 3DO to monitor body composition changes in remote areas that do not have access to traditional body composition modalities. These results highlight the capabilities of 3DO as a feasible method to assess body composition that can provide useful feedback of body composition and change so countermeasures and interventions can be implemented in a timely manner.

To date, there has been limited studies that examined possible methods to assess body composition with simulated microgravity. Zahariev et al.^22^ used skinfold thickness measurements to estimate percent fat (%fat) compared to %fat by oxygen-18 labeled-water as the criterion. Participants were measured during the control period and after 6 days of head down bed-rest (HDBR) at −6°. The control period had a strong agreement (*R*^*2*^ = 0.99) between the two methods. After the HDBR period, the relationship was weakened for both males and females (*R*^*2*^ = 0.67 and 0.77, respectively). Bartok et al.^23^ used bioelectrical impedance spectroscopy (BIS) to compare water volume to magnetic resonance imaging (MRI) muscle volume for calf and upper arm after 3 days of HDBR. They reported MRI muscle volume was on average 15 and 14% lower compared to BIS for arm and calf, respectively, but had strong correlations (*r* = 0.96 and *r* = 0.93, respectively). Although there has been attempts to estimate or find surrogates for body composition in simulated microgravity, the reported values were not as comprehensive as all the metrics reported in this present study. The studies were performed 17 years ago, which shows the difficulty in developing viable methods to assess body composition suitable for challenges met in space. Now, advances in 3DO have the potential to make this possible.

Previous research has indicated that expeditioners generally remain weight stable, with changes ranging up to 2 kg^5^. Our study involved individuals who reported significant increase to their physical activity habits (data not shown), thus larger weight loss was expected. Despite the large weight changes by some individuals, most participants remained within 1 kg of their starting weight throughout the study period. Prior studies have monitored changes in body composition pre- and post-campaigns, relying on BMI as surrogate of change in body composition^24^. Maciejczyk et al. (2016) observed a significant increase in FFM resulting from increased physical activity in their study sample. However, most studies show relative weight stability concomitant to decreases in physical activity^24–26^. The findings by Macijczyk et al. (2016) as well as those in this analysis show that significant changes in body composition can occur in a short period of time as a result of changes to normal dietary and physical activity behaviors. The conclusion that we were able to obtain reliable scans using 3DO assessment in a field setting supports the use of this method to assess body composition in medical checkups in these remote environments.

The benefits of optical imaging, beyond the accuracy and precision shown here, include its ease of access and collection. Young et al.^8^ developed an algorithm to estimate anthropometric measures from two-dimensional (2D) images (photographs). Their algorithm was tested on nine participants that were onboard the International Space Station (ISS). Although the goal of the study was to show stature changes during long-duration spaceflights, the 2D data could potentially be used for body composition. Tian et al.^18^ developed an algorithm that converts 2D images into 3D meshes and predicted accurate and precise total and regional body composition with respects to DXA^27^.

**Fig. 1:**
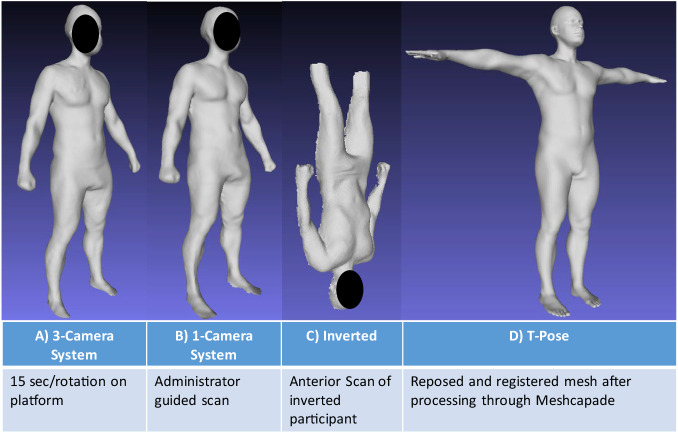
Example 3D meshes from our different scanner protocols. **A** 3D mesh from the SRL-3, **B** 3D mesh from the SRL-1, **C** inverted 3D mesh with artifacts removed and cleaned, **D** 3D mesh in T-pose position after registration with Meshcapade.

Although the partial scans were previously shown to be accurate to whole-body 3DO scans^28^, the current results presented were not entirely equivalent. Several factors should be considered regarding the scan acquisition. In the previous analysis, the preliminary data were from the Fit3D and participants were scanned standing upright. The back-half of the scans were manually removed before templating and reposing with Meshcapade. In the present analysis, participants were scanned suspended upside-down to simulate fluid redistribution. The posterior side was not scanned and the lower legs were removed before analysis. Based on the regional removal analysis (Supplementary Table 1), the main contributing factor appears to be the shape changes and the fluid redistribution. Other than swelling of the upper torso and face area, the abdomen area was sucked in as seen in Fig. 1. Generally, adiposity shape features are seen around the abdomen. Since the abdomen was tucked away, that shape features may have influenced the PCs, resulting in inaccurate FM estimates.

The studies mentioned previously used HDBR with −6° tilt^22,23^. In this current study, participants were hung 90° to the ground. Future studies could evaluate 3DO and the effects of HDBR with a less extreme inversion. Although the agreement could have been influenced by movement and the fluid redistribution, the inverted results remained highly correlated to the SRL-3 and the Fit3D, which means it may need specific calibration or adjustments to resolve.

The total fat and regional fat precision of the inverted scans had a higher %CV and RMSE compared to the SRL-3 and Fit3D. However, the total fat-free and regional fat-free precision were comparable to the SRL-3 and Fit3D. Since FFM is the source of atrophy in astronauts, it is promising to see strong precision in both laboratory and field conditions. The least significant change (LSC) can be calculated from the precision error (Equation 1: LSC = 2.77 x precision error). The LSC is the minimum change needed to have 95% confidence that the change was not due to chance^29^. With the promising precision of FFM, 3DO may be used to monitor change over time^30^.

Compared to previous 3DO studies, the SRL-3 scanner performed similarly when compared to DXA. In this study, total FM, total FFM, and VAT achieved an *R*^2^ of 0.76, 0.97, and 0.78 with an RMSE of 2.62, 2.04, and 0.06 kg, respectively. Ng et al.^13^ reported (in a healthy sample) an *R*^2^ of 0.88, 0.93, and 0.67 with an RMSE of 3.38, 3.38, and 0.16 kg for total fat mass, total fat-free mass, and VAT, respectively, in males and similarly in females. Wong et al.^20^ reported an *R*^2^ of 0.90, 0.95, and 0.78 with an RMSE of 2.99, 2.9, and 0.13 kg for total fat mass, total fat-free mass, and VAT, respectively, in males and similarly in females. This showed how 3DO can be robust across different systems. The *R*^2^ for total FM reported in this study was lower compared to the other two studies, but that can be attributed to the reduced total FM range in this sample^31^. Total FM ranged from 7.3 – 26 kg, while it ranged from 6.1 to 72.7 kg in the other two studies.

If this prototype were to be tested in space (i.e., ISS), the ideal configuration would have the cameras in the framework of the ship (i.e., the wall) and connected to a computer that could be mounted to the wall as well to conserve space. Astronauts would be able to scan themselves either with a full rotation or a partial scan of the anterior side of their body. They would not need to position themselves in any particular pose as Meshcapade can repose their mesh afterwards. Following, internal algorithms would process the scans and provide real-time body composition results. Astronauts can scan themselves weekly, daily, or even several times a day to improve the precision because there are no harmful side-effects of 3DO. In this on-going effort, additional field-studies are being conducted in Antarctica to access the user-friendliness of these systems and data quality obtained from crew members. A recent study utilized total body water (TBW) in addition to 3DO imaging to create a multi-compartment model that improved accuracy and sensitivity to body composition changes^32^. We plan to evaluate accessible wearables that can estimate TBW^33^ coupled with estimates from 3DO to enhance the assessment for astronauts and those in remote environments.

One strength of this study was the various metrics studied. Not only were total body composition values reported but regional values as well. Furthermore, we were able to test our laboratory methods in a field setting, which suggests 3DO can be used outside controlled laboratory settings with proper training and testing protocols. However, the study is not without limitations. The study sample was small and not ethnically diverse as it was a small pilot study. Fluid redistribution by microgravity was simulated with inversion, which is an extreme shape change compared to the common 6-degree HDBR or microgravity changes observed during spaceflight. Additionally, the gravity boots and squat rack limited the scan view of the inverted meshes. A future study with 6-degree HDBR using 3DO and DXA may address the limitations seen in this study.

Overall, 3DO was accurate and precise when estimating FFM even with fluid redistribution. The Antarctic arm highlighted 3DO’s feasibility in isolated confined environments and success with minimal and remote training. Upon further study and evaluation in microgravity, 3DO may be a suitable body composition modality for longer duration space missions such as Lunar and Mars. 3DO provides a better screening option to track the progress of countermeasures to muscle wasting compared to previously studied methods.

## Methods

### Study design

The study consisted of cross-sectional and prospective arms. Cross-sectional data (ASTRO) were collected in a laboratory setting to examine device accuracy and precision using simulated conditions of microgravity and isolation. Duplicate 3DO scans were acquired while standing and while inverted using gravity boots. Duplicated whole-body DXA scans were acquired as the criterion body composition measurement. The prospective study arm (ABCS) collected during an Antarctic research expedition on a sample of expeditioners and was designed to assess the challenges of remote scanning in addition to understanding the effects of changes in environmental, diet, and physical activities on body composition. Duplicate 3DO scans were acquired using a mobile 3DO camera mounted to a laptop on a stationary participant. Participants in both arms received measures of height and weight as well as anthropometry.

### Participants

The recruitment goal of ASTRO was 30 participants (15 male) from the Honolulu community surrounding the University of Hawaii Cancer Center (UHCC). Participants were prescreened by phone and deemed ineligible if they were pregnant, breastfeeding, had missing limbs, were unable to stand unassisted for <1 min, or unable to lay still for <3 min.

The recruitment goal for ABCS was 30 participants (22 male) from expeditioners at the Australian Antarctic Division’s Davis and Mawson research stations in East Antarctica. Participants were directly recruited by the single medical practitioner based at each station. The assessments occurred during, or around, routine monthly medical appointments. Body composition change was quantified via 3DO scanning, but results were not provided to study participants. Medical practitioners were trained remotely to use the system to collect triplicate scans to quantify precision and barriers and enablers of scanning in a field-setting. ABCS participants did not undergo a DXA scan due to inaccessibility in Antarctica.

All participants had height and weight measured to the nearest 0.1 cm and 0.01 kg, respectively, using either a SECA 274 (ASTRO) or 264 (ABCS) stadiometer/digital scales (SECA GmbH, Hamburg, Germany). All participants provided informed consent and the study protocols were approved by the UH Institutional Review Board (IRB #2019-00578, ASTRO) and the University of Tasmania Human Research Ethics Committee, (Project #24377, ABCS).

### Dual-energy X-ray absorptiometry

ASTRO participants located in regions other than Antarctica underwent a single whole-body DXA scan using a Hologic Discovery/A system (Hologic Inc., Marlborough, Massachusetts) according to International Society for Clinical Densitometry guidelines^34^. The Hologic block phantom was scanned daily for calibration. All scans were performed in whole-body mode and analyzed by a single certified technologist using Hologic Apex version 5.6 with the National Health and Nutrition Examination Survey Body Composition Analysis calibration option disabled.

### 3D optical imaging

ASTRO and ABCS participants changed into form-fitting tights, a sports bra without underwire if female, and a swim cap. Some ABCS participants reported to have used thermal tops and pants with the swim cap, others wore only underwear and a swim cap. Expeditioners who had shaved heads did not wear the swim cap. Duplicate scans, with repositioning between each scan, were taken on each 3DO system in the laboratory setting or on the candidate system in the field arm. Each system outputs a 3D mesh that consist of vertices and faces.

#### Fit3D ProScanner

ASTRO participants underwent duplicate 3DO scans using the Fit3D ProScanner version 4.x (Fit3D Inc., San Mateo, California) as the criterion 3DO scans. Participants grasped telescoping handles on the scanner platform and stood up straight with shoulders relaxed and arms positioned straight and abducted away from the torso. The platform rotates once around and takes ~45 s for the completion of the scan. Final point clouds were converted to a mesh connected by triangles with ~300,000 vertices and 600,000 faces to represent body shape^18^. All 3DO scans taken on the Fit3D ProScanner were transferred to Fit3D Inc., who securely transferred the data to UHCC for statistical analysis.

#### 3-camera scanner, SRL-3

**Fig. 2:**
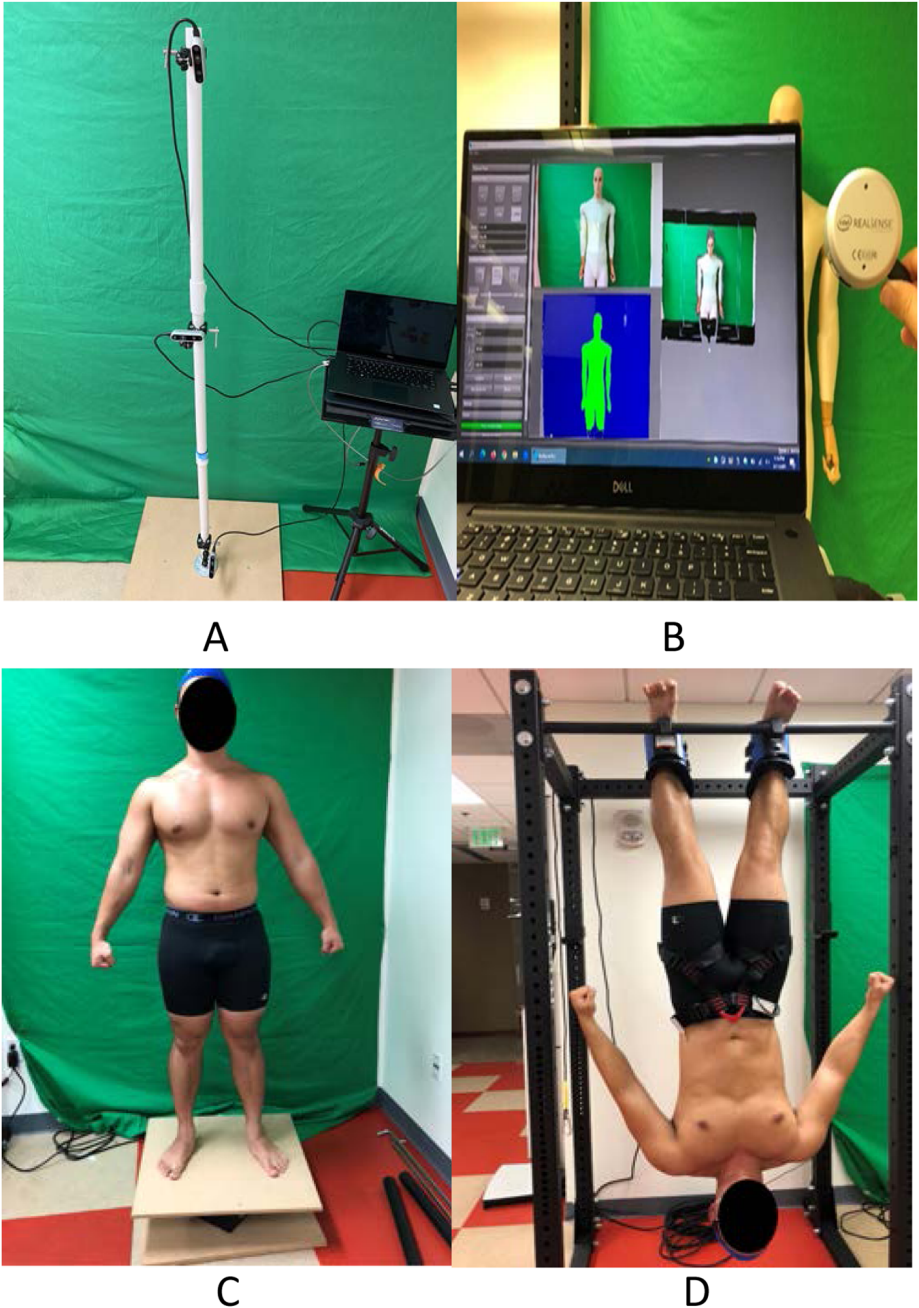
ASTRO data collection setup for standing and inverted scans. **A** the SRL-3 system consisting of three Intel RealSense Depth Cameras D435, **B** the SRL-1 system consisting of one Intel RealSense LiDar Camera L515, **C** participant standing on the rotating platform in an A-pose, **D** same participant inverted with gravity boots in an A-pose.

A 3-camera custom scanner, SRL-3 (Fig. 2A) was designed to simulate scanning in microgravity with the cameras to mounted inside the space capsule and the astronaut freely rotating while being scanning themselves. The SRL-3 utilized three stationary Intel RealSense Depth Cameras D435 (Intel Corp., Santa Clara, California), a Dell XPS 15 Touch Laptop (Dell Inc., Round Rock, Texas), and RecFusion Pro software (version 1.4.7, ImFusion GmbH, Munchen, Germany). The cameras were mounted vertically using a PVC pipe spaced 28 cm from each other, 66 cm away from the participant, and angled at 45 degrees downwards (top camera), 0 degrees (middle camera), and 25 degrees upward (bottom camera) to maximize the resolution and coverage of the body^35^. Prior to scanning, the cameras were calibrated according to ImFusion’s protocol with a checkered calibration board. Two cameras were calibrated at a time (i.e., cameras 1 and 2; then cameras 2 and 3) with simultaneous view of the calibration board. Next, a rectangular field of view was defined in the software to encompass the whole body. We found the optimum parameter settings to be a depth format resolution of 1280 × 720, frame rate of 30 frames per second (fps), color resolution of 1280 × 720 at 30 fps, and laser power set to max. Participants were rotated on a custom designed turntable consisting of a 61 cm^2^ square wooden platform mounted to a rotating base (TS-650lb, Brussian Strokes Inc., Montville, New Jersey). See Fig. 2C. Footprint decals on the platform were used to standardize positioning. Image capture was done with live reconstruction and three different rotation speeds, 2, 4, and 7.5 revolutions per minute [rpm] for total rotation times of 8, 15, and 30 s.

To simulate fluid redistribution, analogous to space, participants were scanned while hung upside down on a squat rack (Fig. 2D), RML-390F Flat Foot Monster Lite Rack (Coulter Ventures, LLC., Columbus, OH, USA). Gravity boots were used to suspend the participant from their ankles. For safety purposes, two lab personnel were present to assist the participants getting on and off the rack. The SRL-3 was placed on a rolling dolly. Scanning began on the participant’s left side and ended at the right side with 5-s dwell times on the left, middle, and right positions. Scans were taken using the same parameters previously mentioned. However, with the limited time participants could be hung upside down and limited range of the dolly, only the anterior side of the body was imaged.

After all scans were acquired, raw scans were prepared for analysis by manually removing artifacts captured in every mesh using Meshlab 1.3.2 (Consiglio Nazionale delle Ricerche, Rome, Italy). Artifacts included the platform, floor, rack, gravity boots, etc. Note that the removal of the gravity boots removed the lower legs of the participants from the raw mesh.

#### 1-camera scanner, SRL-1

A second custom one-camera system, SRL-1 (Fig. 2B) was implemented to evaluate the performance of a system that is simpler in design but requires a helper to acquire the scan. This system used a single Intel RealSense LiDAR Camera (L515, Intel Corp., Santa Clara, California), a laptop as described in the 3-camera system, and ImFusion software, version 2.1.0. The depth camera’s resolution is influenced by the distance from the object, while LiDAR cameras can maintain higher resolution over a greater distance. For SRL-1, LiDAR technology was used since its resolution is not related to the distance away from the scan object as is the case for standard stereo depth cameras^36,37^. Being a 1-camera system, no calibration images were taken. Other imaging settings were the same as SRL-3. To scan, the examiner walked around a stationary participant while holding the laptop with the camera attached at a rate of about 30 sec per scan, moving the camera up and down to capture the entire body until one-revolution was completed. This method of scanning was used by the participants at Davis and Mawson Stations in Antarctica.

### 3D optical scan analysis

All 3DO meshes were sent to Meshcapade (Meshcapade GmbH, Tubingen, Germany) for templating and to be digitally reposed. Their algorithm registers each mesh to a 110,000-vertex mesh with full anatomical correspondence. This means each vertex corresponds to a specific anatomical location across all registered meshes and are no longer random. All meshes were digitally reposed to a *T*-pose, where the person was standing straight, arms were brought horizonal and in plane with the body, and arms and legs were straightened. For the inverted meshes, which only had the anterior side, Meshcapade’s algorithm predicted the missing posterior side with their shape model. In general, when the template is registered to a target mesh, the template fits to the topology of the target mesh by optimizing error functions for the pose and shape, separately. If the target mesh is missing certain areas, the template, which is a “water-tight” mesh, fits to those missing areas based on the available shape information from the target mesh^20,38^.

The standing 3DO meshes were compared to the inverted meshes (ASTRO). However, the inverted meshes did not have the posterior side of the body or the lower legs due to the gravity boot artifacts. Therefore, the posterior side and lower leg regions were predicted by Meshcapade’s algorithm to render a complete mesh. To test if our observed differences were caused by fluid shift shape changes or the predicted parts of the mesh, an intermediate analysis was performed on the standing meshes with parts of it removed manually in Meshlab. Specific regions (i.e., back, legs, lower legs, head/neck, and arms) were also removed individually to evaluate if a specific area may lead to differences. This will be referred to as the regional removal analysis for this paper.

### Statistical analysis

All templated meshes were transformed into principal component (PC) space using an established shape model. 3DO body composition estimates were derived using published equations built off the shape model^20^. In ASTRO, these 3DO estimates were compared to DXA by linear regression. Accuracy was reported with the coefficient of determination (*R*^2^) and root mean square error (RMSE). Duplicate scans from both study arms were used to evaluate the test-retest precision also known as short-term precision. This was reported with the percent coefficient of variation (%CV) and RMSE^39^. Student’s *t*-test determined mean differences (*p*-value < 0.05). This analysis was done in R version 4.2.1 (R Core Teams).

For ABCS, changes in body composition were calculated as an absolute change for each participant, while the average overall change and percentage of change (compared to baseline) were reported, including minimum and maximum changes for each measure of body composition. This analysis was performed using SAS version 9.4 for Windows (SAS Institute, Cary, NC, USA).

### Supplementary information

Supplementary Information

## Data Availability

Data described in the manuscript contains protected health information and will be made available upon request pending researchers who meet the criteria for access to confidential data.

## Code availability

Code book and analytic code is available on Github: https://github.com/shepherd-lab/body-shape-evaluation.
