# Supplementary material for: Evaluation of body shape as a human body composition assessment in isolated conditions and remote environments
Source: NPJ Microgravity. 2024 Jun 24;10:72. doi: 10.1038/s41526-024-00412-5 (PMC11196706; doi:10.1038/s41526-024-00412-5)
Supplement: Supplementary file 1 — Supplementary Information [file 41526_2024_412_MOESM1_ESM.pdf]

# Evaluation of Body Shape as a Human Body Composition Assessment in Isolated Conditions and Remote Environments

Michael C. Wong[1], Jonathan P. Bennett[1], Lambert Leong[1], Yong E. Liu[1], Nisa N. Kelly[1], John Cherry[2], Kate Kloza[2], Bosco Li[2], Sandra Iuliano[3], Jean Sibonga[4], Aenor Sawyer[5], Jeff Ayton[2], John A. Shepherd[1]

[1]Department of Epidemiology, University of Hawaii Cancer Center, Honolulu, Hawaii, USA; [2]Polar Medicine Unit, Australian Antarctic Division, Kingston, AUS; [3]Departments of Medicine and Endocrinology, Austin Health, University of Melbourne, Melbourne, Australia; [4]National Aeronautics and Space Administration Johnson Space Center, Houston, Texas, USA; [5]University of California, San Francisco, California, USA;

Corresponding Author. Email: johnshep@hawaii.edu

Supplementary Table 1. Regional removal analysis (n=28)

| | Reference | | Inverted | | All[1] | | Arms[2] | | Back[2] | | Head[2] | | Legs[2] | | Lower Legs | |
|---|---|---|---|---|---|---|---|---|---|---|---|---|---|---|---|---|
| | $R^2$ | RMSE | $R^2$ | RMSE | $R^2$ | RMSE | $R^2$ | RMSE | $R^2$ | RMSE | $R^2$ | RMSE | $R^2$ | RMSE | $R^2$ | RMSE |
| Total Fat | 0.74 | 2.69 | 0.50 | 2.87 | 0.67 | 2.19 | 0.73 | 2.75 | 0.76 | 2.44 | 0.75 | 2.70 | 0.78 | 2.32 | 0.72 | 2.87 |
| Total Lean | 0.97 | 2.03 | 0.94 | 2.86 | 0.96 | 2.31 | 0.97 | 2.00 | 0.97 | 2.17 | 0.97 | 2.06 | 0.98 | 1.87 | 0.96 | 2.23 |
| Percent Fat | 0.69 | 3.52 | 0.54 | 3.26 | 0.71 | 2.68 | 0.70 | 3.51 | 0.72 | 3.43 | 0.70 | 3.60 | 0.77 | 2.94 | 0.64 | 3.93 |
| VAT | 0.76 | 0.06 | 0.36 | 0.11 | 0.68 | 0.07 | 0.75 | 0.07 | 0.67 | 0.07 | 0.76 | 0.07 | 0.73 | 0.08 | 0.72 | 0.08 |
| Arm Fat | 0.50 | 0.27 | 0.19 | 0.30 | 0.31 | 0.25 | 0.52 | 0.27 | 0.51 | 0.26 | 0.49 | 0.29 | 0.48 | 0.27 | 0.48 | 0.3 |
| Arm Lean | 0.93 | 0.32 | 0.92 | 0.35 | 0.91 | 0.32 | 0.94 | 0.29 | 0.94 | 0.29 | 0.94 | 0.29 | 0.94 | 0.28 | 0.94 | 0.29 |
| Leg Fat | 0.58 | 0.59 | 0.15 | 0.76 | 0.49 | 0.53 | 0.58 | 0.62 | 0.66 | 0.58 | 0.59 | 0.61 | 0.65 | 0.48 | 0.59 | 0.63 |
| Leg Lean | 0.92 | 0.62 | 0.86 | 0.65 | 0.88 | 0.64 | 0.91 | 0.56 | 0.91 | 0.64 | 0.93 | 0.55 | 0.91 | 0.58 | 0.90 | 0.64 |
| Trunk Fat | 0.80 | 1.25 | 0.57 | 1.38 | 0.68 | 1.15 | 0.76 | 1.42 | 0.81 | 1.04 | 0.80 | 1.28 | 0.79 | 1.28 | 0.78 | 1.34 |
| Trunk Lean | 0.94 | 1.49 | 0.93 | 1.27 | 0.93 | 1.46 | 0.94 | 1.38 | 0.94 | 1.43 | 0.95 | 1.34 | 0.94 | 1.32 | 0.94 | 1.41 |

SRL-3 scanner was used for this analysis.

[1] Refers to the standing scan from the SRL-3 with all regions removed to mimic the inverted scan but without inversion.

[2] Only the posterior side of this body part was removed before processing through Meshcapade.

Supplementary Table 2. Comparison of automated anthropometry of region removal analysis to SRL-3 reference mesh.

| | | $R^2$ | RMSE | Mean Difference (cm) | P-Value |
|---|---|---|---|---|---|
| Head | | | | | |
| | Neck Circ. | 0.99 | 0.26 | -0.42 | **<0.001** |
| | Waist Circ. | 0.99 | 0.10 | -0.04 | 0.080 |
| | Hip Circ. | 0.99 | 0.11 | -0.05 | 0.050 |
| | Thigh Circ. | 0.99 | 0.09 | -0.03 | 0.151 |
| | Arm Circ. | 0.99 | 0.23 | -0.01 | 0.911 |
| | Chest Circ. | 0.99 | 0.10 | -0.07 | **0.009** |
| Arms | | | | | |
| | Neck Circ. | 0.99 | 0.09 | 0.06 | **0.048** |
| | Waist Circ. | 0.99 | 0.14 | -0.05 | 0.168 |
| | Hip Circ. | 0.99 | 0.17 | 0.04 | 0.399 |
| | Thigh Circ. | 0.99 | 0.10 | 0.00 | 0.871 |
| | Arm Circ. | 0.95 | 1.05 | -0.83 | **0.038** |
| | Chest Circ. | 0.99 | 0.17 | 0.17 | **0.002** |
| Legs | | | | | |
| | Neck Circ. | 0.99 | 0.08 | 0.04 | 0.086 |
| | Waist Circ. | 0.99 | 0.21 | -0.12 | **0.028** |
| | Hip Circ. | 0.94 | 1.29 | -0.73 | 0.107 |
| | Thigh Circ. | 0.95 | 0.70 | -0.82 | **0.017** |
| | Arm Circ. | 0.99 | 0.49 | -0.5 | **0.002** |
| | Chest Circ. | 0.99 | 0.15 | 0.01 | 0.891 |
| Back | | | | | |
| | Neck Circ. | 0.99 | 0.19 | 0.02 | 0.754 |
| | Waist Circ. | 0.99 | 0.86 | 0.49 | 0.166 |
| | Hip Circ. | 0.99 | 0.16 | 0.02 | 0.649 |
| | Thigh Circ. | 0.99 | 0.14 | -0.06 | 0.139 |
| | Arm Circ. | 0.99 | 0.62 | -0.32 | **0.043** |
| | Chest Circ. | 0.99 | 0.99 | 1.37 | **0.007** |
| Lower Legs | | | | | |

| | | | | |
|---|---|---|---|---|
| Neck Circ. | 0.97 | 0.50 | -0.1 | 0.461 |
| Waist Circ. | 0.99 | 0.99 | -0.13 | 0.616 |
| Hip Circ. | 0.99 | 0.29 | 0.03 | 0.684 |
| Thigh Circ. | 0.97 | 0.73 | -0.05 | 0.765 |
| Arm Circ. | 0.71 | 3.52 | 0.81 | 0.343 |
| Chest Circ. | 0.99 | 0.25 | 0.11 | 0.078 |
| All Parts Removed | | | | |
| Neck Circ. | 0.95 | 0.66 | -0.61 | **0.002** |
| Waist Circ. | 0.97 | 1.31 | 0.74 | 0.206 |
| Hip Circ. | 0.96 | 0.89 | -0.28 | 0.567 |
| Thigh Circ. | 0.94 | 0.76 | -0.40 | 0.215 |
| Arm Circ. | 0.87 | 1.54 | -2.55 | **<0.001** |
| Chest Circ. | 0.94 | 1.76 | 0.9 | 0.261 |
| Inverted | | | | |
| Neck Circ. | 0.81 | 1.71 | 0.20 | 0.639 |
| Waist Circ. | 0.94 | 2.01 | -3.13 | **<0.001** |
| Hip Circ. | 0.80 | 1.85 | -2.12 | **0.014** |
| Thigh Circ. | 0.90 | 0.90 | -1.03 | **0.022** |
| Arm Circ. | 0.88 | 1.49 | 1.75 | **0.005** |
| Chest Circ. | 0.97 | 1.39 | 1.53 | **0.034** |

Abbreviations: Circ. (Circumference)
$R^2$ and RMSE reported from linear regression. Mean difference and p-value were reported from paired Student’s t-test.
Mean difference = Experimental measurement – Reference measurement
**Bold**: mean difference was significant ($p>0.05$)

Supplementary Table 3. Agreement between scan speeds on SRL-3 scanner

| Outcome | 8s $R^2$ | 8s RMSE | 30s $R^2$ | 30s RMSE |
|---|---|---|---|---|
| Total Fat | 0.96 | 1.04 | 0.98 | 0.80 |
| Total Lean | 0.99 | 1.04 | 0.99 | 0.80 |
| Percent Fat | 0.94 | 1.52 | 0.97 | 1.17 |
| VAT | 0.88 | 0.05 | 0.94 | 0.03 |

Abbreviations: s (seconds), VAT (visceral adipose tissue)
All outcomes are presented in kg except percent fat.
8s and 30s were compared to the reference scan taken at 15s.
